# Promoting Exercise as a Therapeutic Intervention in Multiple Sclerosis: Barriers, Efficacy, and Social Prescribing Strategies

**DOI:** 10.26502/fccm.92920478

**Published:** 2026-01-07

**Authors:** Kristen Mittl, Devendra K. Agrawal

**Affiliations:** 1 Department of Translational Research, College of Osteopathic Medicine of the Pacific, Western University of Health Sciences, Pomona, California 91766 USA; 2 College of Osteopathic Medicine of the Pacific – Northwest Western University of Health Sciences, Lebanon, Oregon, 97355 USA

**Keywords:** Aerobic exercise, Barriers to exercise, Expanded disability status scale, Multiple sclerosis (MS), People with multiple sclerosis (pwMS), Physical disability, Psycho-social status, Social prescribing

## Abstract

Multiple sclerosis (MS) is a chronic neurologic disease associated with significant physical, cognitive and social burden. Substantial evidence supports the safety and benefits of aerobic exercise for people with MS (pwMS), including improvements in fatigue, mobility, cognition, mood and quality of life. While exercise is recommended across all disability levels in clinical guidelines, real-world adherence remains low. This narrative review summarizes the benefits of aerobic exercise in MS and examines the symptom-related, psychosocial, socioeconomic, and healthcare system barriers that limits its implementation. These barriers reflect the broader influence of social determinants of health, which play a critical role in MS outcomes yet remain under-addressed in intervention research. We highlight social prescribing, a patient-centered approach that connects individuals to non-medical, community-based resources to address social needs, as a strategy to reduce barriers to exercise participation for pwMS. Future research should focus on developing and evaluating MS-specific interventions to improve exercise adherence and promote equitable access to physical activity to improve health outcomes.

## Introduction

Multiple sclerosis (MS) is a chronic, inflammatory, demyelinating disease of the central nervous system, with focal white matter lesions defining its pathology [1]. MS is the most common cause of nontraumatic disability in young adults [2]. The most recent reliable prevalence of MS study in the United States reported that the estimated prevalence of MS in 2010 was over 700,000 cases – the highest reported MS prevalence to date [3]. The disease course is defined by relapses, episodes of acute neurological worsening that then lingers for days or weeks, followed by some degree of recovery, and progression [4]. Even within the same MS subtype, disease course is highly heterogeneous, ranging from very mild forms with minimal disability after more than 20 years to rapidly progressive forms in which significant disability develops within only a few years [5]. Fatigue, problems with walking and balance, depression, and learning and memory impairment are amongst the most common and burdensome symptoms that occur across MS subtypes [6]. As there is no known cure for MS, treatment focuses on alleviating symptoms and improving quality of life, with additional effective approaches on top of disease modifying therapies including interventions such as physical activity and balance-based therapies [7].

Among these, aerobic exercise has emerged as one of the most effective and versatile interventions. Decades of evidence demonstrate that aerobic exercise is safe for people with MS (pwMS), with vast benefits including improved physical functioning, cognition, fatigue, and quality of life [8,9]. Since exercise is considered a beneficial tool in mitigating disease symptoms, it is recommended across all levels of disability in MS clinical care guidelines. Despite well-established benefits, research consistently shows that pwMS engage in lower levels of physical activity than healthy individuals and are less likely to exercise regularly, facing distinct barriers and motivational challenges that further limit their participation [10]. Given the multifactorial nature of the barriers to exercise, and their interplay with social determinants of health, pwMS face complex obstacles that make meeting exercise recommendations nearly impossible. One promising approach is social prescribing, a model of linking patients with sources of support within the community via a non-medical referral to improve their health and well-being [11]. While social prescribing has shown effectiveness in chronic condition management, its application to neurological diseases, including MS, have not been well studied. Given the well-documented barriers to exercise in MS, this model may provide a novel pathway to support patients in overcoming obstacles to physical activity.

This narrative review synthesizes current evidence on the benefits of aerobic exercise for pwMS, the barriers that hinder its utilization to improve quality of life outcomes, and the potential role of social prescribing in addressing these challenges. In doing so, it identifies key gaps in the literature and outlines priorities for future research aimed at translating evidence-based recommendations into sustainable, equitable, and patient-centered care.

## The Role of Exercise in Multiple Sclerosis, Aerobic Exercise, and Current Guidelines

### The Role of Exercise in Multiple Sclerosis, Aerobic Exercise, and Current Guidelines

Over the past few decades, there has been a movement in MS research to explore the potential effects of exercise on disease progression, quality of life, and symptom management. The complexity of the disease does not end with its etiology and diagnosis – the symptom presentation is also vast and variable – making the effects of exercise on disease management difficult to study and generalize. Fatigue, however, stands out as a symptom that affects most MS patients. Ramirez, et al. [12] reviewed 54 studies and found that fatigue prevalence in pwMS ranged from 36.5-78.0%. Since fatigue is one of the most highly cited symptoms, researchers investigated the effect of exercise on fatigue management in people with MS.

Several studies found that exercise significantly reduced fatigue in pwMS. For example, Razazian and colleagues [13] reviewed 31 clinical trial studies implementing various exercise programs and found a significant reduction in fatigue severity post-intervention, suggesting that physical exercise may reduce fatigue in pwMS. The authors highlight the unique mechanism of aerobic exercise, as the high consumption of oxygen during this activity positively influences metabolic pathways even when the musculoskeletal system is inactive, reducing the duration of oxidation, and important factor in fatigue and sluggishness. However, not all studies find this significant finding, and the authors note this may be due to the difficult nature of studying fatigue, its influence by many other factors, and, for this study where mostly Iranian clinical trials were reviewed, the results could be due to the younger population of the country, suggesting a larger percentage of younger patients in the intervention group compared to the demographics of other studies, therefore skewing the results. If this is true, however, it would suggest age as another factor that could impact the efficacy of exercise in MS fatigue management. In another review, Moss-Morris et al. performed a meta-analysis of 13 exercise interventions from 10 studies, which suggested at the end of treatment, exercise on average has a moderate to large effect on fatigue [14]. Overall, exercise seems to be a promising tool for lessening fatigue in pwMS, once obstacles for this population are addressed, which we will focus on in this review.

### Evidence and benefits of aerobic exercise in multiple sclerosis

Now, to focus on aerobic exercise, a randomized control trial investigated the effect of progressive aerobic exercise (PAE) on fatigue and walking in pwMS [15]. They found that 24 weeks of supervised PAE resulted in a clinically meaningful reduction in total fatigue impact scores but only small improvements in walking ability and capacity. Massot et al. [16] conducted a multicenter randomized control trial to evaluate the effectiveness of aerobic treadmill training on walking distance in pwMS. They found significant improvements in walking distance, speed, and balance, but an absence of improvement in fatigue. Both studies present evidence to support aerobic exercise as a well-tolerated and beneficial intervention for pwMS, and their differing positive outcomes highlight the uniqueness of exercise protocols even under the umbrella of aerobic exercise.

Aerobic exercise may also improve cognitive and emotional symptoms associated with MS. Given the mounting evidence for the “neurogenerative” potential of exercise in healthy people, Stellmann et al. [17] conducted a randomized control trial to see how aerobic exercise impacts functional and structural CNS reorganization in MS and found an increase in connectivity after 3 months of moderative aerobic exercise. Another randomized control trial found that in both the control group (pwMS + coordination exercises) and study group (pwMS + coordination exercises + aerobic exercise) depression decreased after 6 weeks, but the decrease was greater in the study group, suggesting the hormone irisin, released from skeletal muscle during exercise, is a key player in the puzzle of aerobic exercise and symptom management [18]. These studies and many others showcase the potential of aerobic exercise in MS symptom alleviation.

### Current guidelines and recommendations for exercise in multiple sclerosis

**Figure 1:**
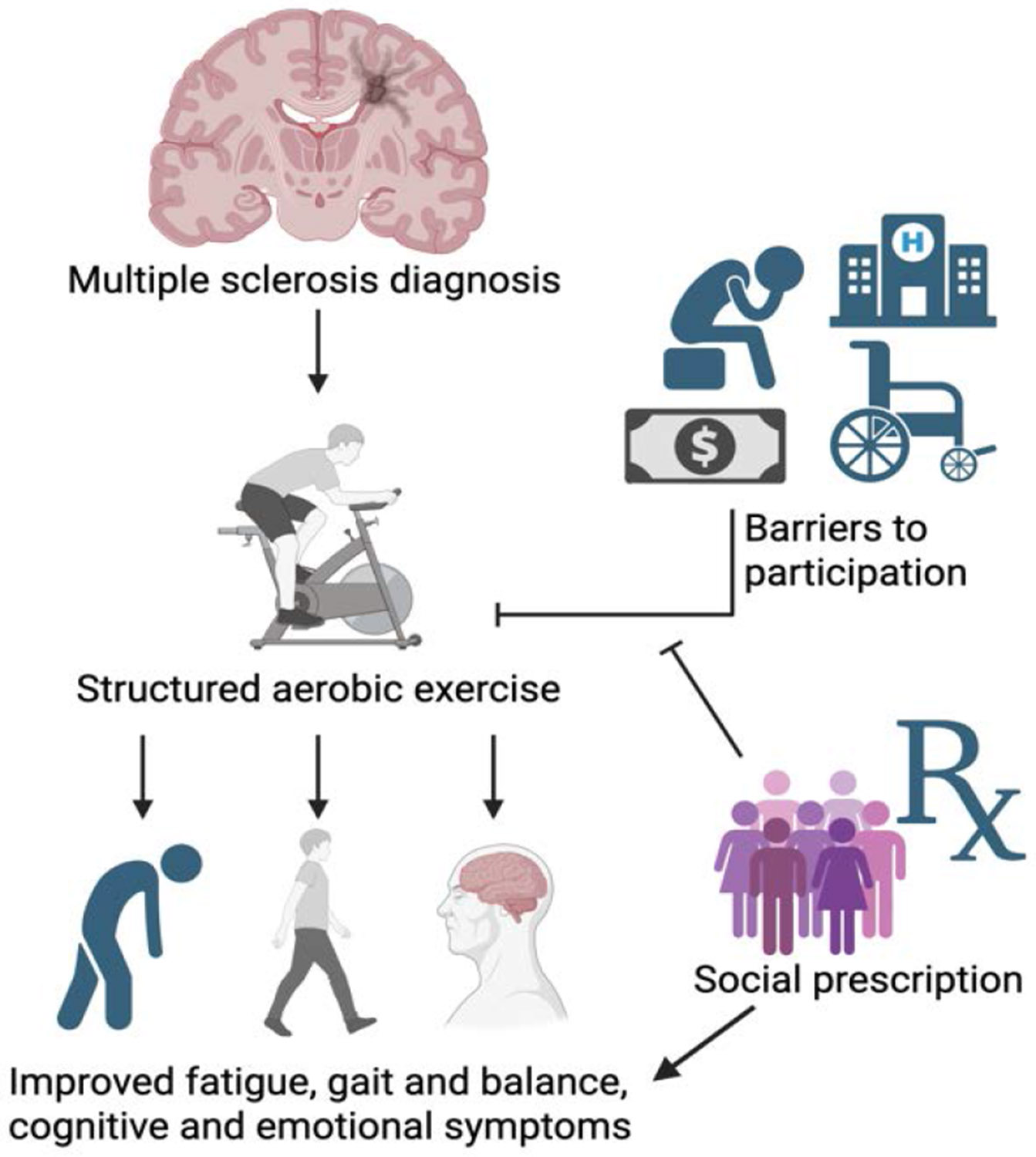
Schematic diagram showing the structured aerobic exercise improving fatigue, gait and balance, cognitive and emotional symptoms, and the potential negative effects of barriers to participation or negative/positive effect of social prescription.

As research investigating – and increasingly suggesting – those individuals with MS benefit from exercise through improved quality of life outcomes has mounted, the need to educate clinicians on evidence-based exercise recommendations has become urgent. In response, the National Multiple Sclerosis Society collaborated with field experts in 2020 to publish exercise and lifestyle guidelines tailored to disability levels ranging from 0 to 9.0 on the Expanded Disability Status Scale (EDSS) [19]. For individuals with EDSS scores of 0-4.5 and 5.0-6.5, the recommendations include aerobic, resistance, flexibility, and neuromotor training. In the latter range, referrals to specialists are emphasized to ensure safety and prevent overexertion as disability progresses (Figure 1).

Notably, for EDSS categories 7.0-7.5, 8.0-8.5, and 9.0, the guidance is primarily based on expert opinion, with limited published data available. At these higher levels of disability, exercise recommendations shift to focus on extremity mobility, respiratory function, and flexibility, with increasing reliance on specialized equipment and caregiver support. The Society emphasizes that “healthcare providers should endorse and promote the benefits/safety of exercise and lifestyle physical activity for every person with MS.” While these recommendations mark significant progress in integrating exercise into MS care, they also reveal critical gaps in the research particularly regarding exercise in individuals with advanced disability.

## Barriers to Aerobic Exercise Participation in Multiple Sclerosis

### People with multiple sclerosis engage in less physical activity

It is well documented that pwMS are less physically active than their healthy counterparts, despite growing evidence that regular physical activity offers significant benefits for disease management. For example, Casey et al. [20] conducted a meta-analysis involving 198 pwMS and found that their mean daily moderate-to-vigorous physical activity (MVPA) was significantly lower than that of an age-matched comparison group – 18.4 minutes per day versus 27.3 minutes, respectively. Such findings are concerning, particularly given that the general population itself often fails to meet established physical activity (PA) guidelines. Current recommendations advise at least 150 minutes per week of MVPA to promote general health; however, even small increases in activity among people with chronic conditions have been shown to improve cardiovascular and overall health outcomes [21]. While pwMS consistently report lower levels of physical activity, addressing key barriers and promoting structured interventions, such as aerobic exercise, may yield meaningful improvements in both general health and MS-specific symptoms (Figure 1).

### Symptoms of multiple sclerosis can hinder aerobic exercise participation

Fatigue and mobility impairments are among the most reported and debilitating symptoms for pwMS, and more than 75% of individuals with MS will experience significant gait dysfunction during their lifetime [22]. These symptoms are substantial barriers to engaging in aerobic exercise – an activity that can be challenging even for healthy individuals. In a cross-sectional study involving 252 pwMS, 69% were categorized as fatigued, and this group engaged in significantly less MVPA than their non-fatigued counterparts [23]. However, when controlling for clinical disability scores, the differences in MVPA between the groups were no longer statistically significant. This suggests that the higher levels of mobility impairment observed in the fatigued group may play a key role in driving reduced physical activity levels. These findings underscore the complex interplay and symptom stacking common in MS, where co-occurring impairments exacerbate functional limitations. As such, exercise interventions for pwMS with both fatigue and mobility impairments must be tailored differently than for those with isolated or less severe symptoms.

Heat sensitivity is a distinct symptom experienced by many pwMS. Uhthoff’s phenomenon, a well-documented feature of MS, is characterized by a temporary and reversible onset and worsening of symptoms in response to increases in core body temperature [24]. This phenomenon is important to consider in the context of aerobic exercise therapy, as physical activity inherently increases metabolic demand and core temperature, potentially causing intolerable and unpleasant symptoms for pwMS during exercise. In a study by Allen et al., pwMS demonstrated significantly lower whole-body sweat loss during exercise compared to matched healthy controls, suggesting that demyelination may disrupt thermoregulatory pathways and cause pwMS to reach their thermal threshold more prematurely [25]. These findings underscore the importance of designing exercise interventions that minimize heat buildup in this population. Cooling strategies and aquatic-based exercise programs have been explored to mitigate thermally induced symptom exacerbation; however, further research is needed to implement these approaches.

Diurnal and day-to-day fluctuations in symptom severity represent a less apparent yet significant barrier to aerobic exercise participation in pwMS. Prior research has demonstrated that pwMS experience a more rapid rise in fatigue severity throughout the day, with fatigue typically peaking in the late afternoon – earlier than in healthy individuals [26]. In addition, a study examining symptom patterns across consecutive days found that most chronic MS symptoms do not exhibit carryover effects from one day to the next, underscoring the unpredictable nature of the disease. Interestingly, the one exception was fatigue – elevated fatigue one day was associated with lower fatigue the following day [27]. These temporal patterns have important implications for implementing aerobic exercise in this population. For instance, morning exercise may be preferable given the earlier onset of fatigue; however, the timing may be impractical for individuals with morning work obligations. Furthermore, the variability in symptom presentation from day to day necessitates a flexible approach to exercise scheduling – yet such flexibility may conflict with program structures that lack options for rescheduling or cancellations. The inconsistency of symptoms in MS presents a substantial challenge to maintaining a regular aerobic exercise routine and must be considered when designing effective interventions.

### People with multiple sclerosis have unique psychosocial factors that shape exercise behavior

Given the chronic nature of MS and the absence of a known cause or cure, pwMS experience disproportionately high rates of mental health disorders. A recent meta-analysis of 58 studies reported that the prevalence of depression and anxiety among pwMS is markedly higher than in the general population – 31% and 22%, respectively [28]. Despite these concerning figures, and growing momentum to integrate aerobic exercise into MS care, few MS-specific studies have examined how depression impacts exercise adherence. However, findings from related neurological populations offer valuable insight. For instance, a recent cross-sectional study involving stroke survivors found that individuals with moderate depression were 31% less likely to adhere to prescribed exercise programs compared to those without depressive symptoms [29]. Given the shared challenges across neurological conditions, it is reasonable to infer that depression similarly impedes exercise adherence in pwMS. This underscores the importance of incorporating mental health support into comprehensive MS care. Without targeted psychological interventions or the integration of cognitive-behavioral strategies into exercise programs, adherence among depressed pwMS may be significantly compromised (Figure 1).

Beyond mental health concerns, MS symptoms profoundly disrupt daily routines and quality of life, leading to a wide range of psychosocial consequences. Social isolation – defined as “a state in which the individual lacks a sense of belonging socially, lacks engagement with others, has a minimal number of social contacts, and they are deficient in fulfilling and quality relationships” [30] – is commonly reported among pwMS. Symptoms such as fatigue, chronic pain, and physical disability often limit participation in activities that foster social connection. A recent cross-sectional study of 200 pwMS found that 55.5% of participants scored high on measures of social isolation [31]. Despite its prevalence and presumed influence on health behaviors, the direct relationship between social isolation and exercise adherence in pwMS remains underexplored. Still, it is plausible that diminished social connection affects motivation, accessibility, and the sustainability of aerobic exercise engagement in this population. This is particularly relevant given that aerobic exercise often requires structured routines and sustained motivation – both of which may be harder to maintain in the absence of social reinforcement or accountability. Thus, understanding the psychosocial barriers unique to aerobic exercise is essential to improving long-term adherence in pwMS.

At first glance, increasing peer support group participation might appear to be a viable solution for countering social isolation and boosting exercise adherence among pwMS. However, evidence supporting the efficacy of such interventions is limited. A recent study examining individuals with MS and epilepsy found that even those who participated in peer support groups had reduced health-related qualify of life (HRQoL) compared to the general population. Moreover, depression was present in 40% of cases – a known predictor of poor HRQoL [32]. While social isolation and depression are distinct constructs, they often coexist, and both reflect the broader psychosocial burden associated with MS. This suggests that while peer support may offer some benefits, it may not be sufficient on its own to mitigate the psychosocial barriers that hinder exercise adherence in pwMS. Alternative or complementary strategies for reducing depression and social isolation should be considered in the design of exercise-based interventions.

Interestingly, the structure of an individual’s social network may play a more nuanced role in MS management than previously recognized. In a cross-sectional study of 427 adults with MS, researchers found that those with more constrained, close-knit social networks reported greater physical impairment than those with larger and more diverse networks [33]. While physical limitations may influence one’s ability to maintain broader social ties, it is equally plausible that restricted social environments contribute to worse health outcomes. Limited social networks may reinforce unhealthy behaviors and reduce exposure to new ideas or motivations – factors critical for behavior change. In the context of promoting aerobic exercise for pwMS, encouraging the development of broader, more diverse social networks may offer greater benefits than relying solely on MS-specific peer support groups. A more varied social pool could provide stronger reinforcement of healthy behaviors, including regular physical activity, thereby serving as a promising strategy to address social isolation as a barrier to exercise adherence.

### Limited resources and current healthcare structure discourage exercise in the care of multiple sclerosis

Socioeconomic status (SES) significantly influences the onset and progression of MS. Higher income and education levels are associated with lower risks of chronic disease [34], and MS is no exception, making social determinants a key factor in disease management. One multicenter study on over 3,600 pwMS found that socioeconomic deprivation was strongly associated with an increased risk of reaching major clinical disability milestones [35]. Lower SES contributes to a twofold burden: individuals are not only more likely to develop greater disability due to systemic disparities, but they also face more barriers to engaging in aerobic exercise, such as financial strain, limited transportation, and reduced access to specialized programs. This creates a paradox in which those most in need of interventions to slow disease progression are least able to access them due to the very socioeconomic challenges contributing to their condition. Given the growing evidence supporting aerobic exercise to lessen symptoms and potentially slow MS progression, this compounded disadvantage represents a significant public health concern that requires targeted intervention to make aerobic exercise a feasible disease management tool for all groups impacted.

Unfortunately, the healthcare system itself serves as a prominent barrier for implementing aerobic exercise as a reliable and safe tool for MS disease management. In the United States, even the most resilient physicians are at risk of burnout and given the average time of a neurology outpatient visit is only about 30 minutes, the current system does not incentivize or promote clinicians to spend extra time and energy with patients to prescribe exercise as a treatment [36,37]. Additionally, the fee for service healthcare delivery model as seen in the United States rewards high-volume, procedure-oriented interventions over more lifestyle-based, longitudinal treatment approaches [38]. While encouraging a patient to incorporate aerobic exercise into their treatment plan has evidence-based medical advantages, it lacks a clear billable code or prescription key drivers to care in a fee for service healthcare model. Physician burnout, time-constrained neurology visits and the fee for service healthcare model collectively discourage adding regimented aerobic exercise as a treatment modality in MS care.

Logically, lengthening appointment times to allow sufficient time for neurologists to provide lifestyle counseling during routine MS follow-up visits seems like an obvious solution to the care gaps outlined. Richardson et al. [39] interviewed 20 practicing neurologists regarding promotion of exercise among patients in comprehensive MS care and found the most mentioned theme was, not surprisingly, “how do I fit exercise promotion into a patient’s appointment?” However, the following commonly appearing themes all revolved around feeling under-trained, under-equipped, and under-guide-lined to promote exercise for their MS patients. For example, one neurologist reported “I have no tools. I have no training except what I observe… Other than that, I don’t really have good guidelines” [39]. Thus, even if appointment times were lengthened and reimbursement lifestyle services were granted, the lack of standardized training pathways or certification programs in exercise management for MS clinicians remains a major barrier to the safe and evidence-based incorporation of exercise prescriptions into routine neurologic practice.

Looking prospectively, the solution to some of these healthcare barriers for incorporating more personalized treatment regimens, such as aerobic exercise, may not even involve neurologists, but rather primary care physicians (PCPs). One study developed a model to assess the projected supply and demand of MS neurologists and found that the MS neurologist specialty workforce may be sufficient now but predicted shortages by 2035 [40]. As specialist appointments become harder to access, more responsibilities for MS care may shift to the patient’s PCP. Studies have already examined the role of PCPs in encouraging exercise among patients with MS. A multicenter cross-sectional study of pwMS reported that 25.5% received PCP advice to engage in exercise and actually 47.1% exercised, while only 17.0% exercised without having received PCP advise, underlining the key role PCPs can play in addressing sedentary lifestyles [41]. While the PCP workforce is also not expected to meet future patient demand by 2035, the oversupply of primary care nurse practitioners may help buffer this workload [42]. Leveraging PCPs for MS patient exercise prescription and adherence is promising but requires more careful analysis to determine its viability as a solution to the healthcare barriers that impede the integration of lifestyle modifications into long-term chronic disease management.

### Potential Impact of Social Prescribing in Multiple Sclerosis

In this article, we have synthesized the evidence demonstrating the benefits of structured and safe aerobic exercise for pwMS. When incorporated into disease management plans, aerobic exercise can improve fatigue symptoms, gait, cognitive function, and emotional well-being. Despite this evidence base, people with MS remain less physically active than the general population, creating a persistent gap between known benefits and real-world implementation. We next outlined the common barriers to exercise faced by pwMS, including symptom-related challenges, psychosocial factors, and limitations within healthcare systems and resources.

While these obstacles can be categorized into distinct domains, a unifying theme emerges on the influence of social determinants of health (SDOH). The World Health Organization defines SDOH as “the conditions in which people are born, live, work, and age.” Experts emphasize that SDOH exert a profound effect on MS outcomes, and that addressing disability prevention solely through disease-modifying pharmaceutical interventions or isolated social interventions will not maximize patient benefit. Yet, interventions directly targeting the impact of SDOH on MS remain “almost completely lacking” [43]. Advancing neurologic health equity will require dedicated efforts to develop and evaluate feasible strategies that lessen the influence of SDOH on disease outcomes.

### Social prescribing: a promising model

Following the 2008 WHO Commission on the SDH, counties across the globe implemented policies to mitigate adverse SDH and their effects on the health of populations, however the involvement of the health sector itself was not leveraged in most policies [44]. Interestingly, general practitioners in the UK had already been addressing their patients’ SDH by referring them to in-house expert non-clinical services since the late 1990s, and as this model expanded throughout the country, the term “social prescribing” was born [45]. As this social service referral model spready, by 2016 the UK created the international Social Prescribing Network, and by 2018 England rolled out a national plan to reimburse one social prescribing “link worker” for every primary care network in the country, a move that extended access to more than 2.5 million people over 5 years [46].

As the integration of social prescribing expanded across the UK and then eventually was adopted by additional high-income countries including the United States, the need for an operational definition of the technique became evident [47]. According to Muhl et al. [48], social prescribing is a holistic, person-centered approach that links individuals with non-medical community services to address SDH and reduce health inequities. Typically involving collaboration between clinical professionals and non-clinical “connectors” (what the UK termed “link workers”), it empowers individuals through personalized action plans, motivational support, and follow-up, while outcomes are evaluated at individual, service, and community levels [48]. How this looks in action varies based on the country the social prescription occurs in. For example, in the US, assistance interventions typically focus on connecting patients to resources that address basic needs, but in countries with more established basic need safety nets, like in the UK, social prescribing link workers assess patients’ needs and refer them to more robust services, like exercise, arts and crafts or volunteering centers, emphasizing quality of life beyond basic needs [49]. In the UK, the social prescribing itself is free, but any costs associated with the activity may result in a small fee for the patient to pay. Social prescribing is unique in its perceived ability to combat patient loneliness, while improving their wellbeing, confidence and feeling of life purpose [50]. Social prescribing is a unique technique targeting SDH with the potential to change the trajectory of patient health in addition to their regularly scheduled medical appointments and treatments.

### Applying social prescribing to the care of multiple sclerosis

At the core of social prescribing is a link worker creating personalized care plans to fill the care gaps patients have because of their unique situations shaped by their SDH. For pwMS combatting psychosocial, societal, or logistical obstacles preventing their engagement in aerobic exercise, social prescribing could help address these commonly cited barriers pwMS face. For example, a recent study utilized the Barriers to Health Promoting Activities for Disabled Persons scale to assess barriers to physical activity pwMS face and found the less active group reported higher scores for “No one to help me”, and “Lack of support from family/friends” [51]. Additionally, the less active group reported not being sufficiently helped by their families and friends, while the active group generally did not cite a lack of assistance as a major barrier. With social prescribing, pwMS who do not have social support would have a link worker to fill in for this clearly instrumental role of providing both logistical and emotional support to allow the person to engage in the physical activity recommended by the neurologist.

To date, no studies have examined the potential role of social prescribing in MS – whether in evaluating its impact, identifying barriers and facilitators to implementation, or developing tailored interventions for pwMS and their caregivers. Some groups have investigated the impacts of social prescribing for other chronic health conditions. For example, a cohort study analysis of over 8,000 adult patients in the UK National Health System with type 2 diabetes found a social prescription targeting patients’ social needs was associated with improved HbA1c levels, thus bettering clinical outcomes associated with their condition [52]. Another research team reviewed the impact of social prescribing for adults with chronic pain in the UK and found that there is evidence that social prescribing improves health and wellbeing outcomes for this population, but more research is needed on its routes to referral and impacts [53]. Given the chronic nature of MS, it is reasonable to hypothesize that the benefits of social prescribing observed in other chronic conditions may translate to pwMS, underscoring the need for targeted investigation.

Although no studies have yet examined the feasibility or design of a social prescribing protocol specific to MS, evidence from other neurologic conditions suggests potential applicability. For example, in Australia, researchers developed a social prescribing program for individuals with cerebral palsy by directly surveying 200 pediatric patients and caregivers to identify unmet social needs, thereby creating an intervention responsive to the unique challenges of this population [54]. They will next perform a pilot randomized controlled trial to further evaluate the intervention among families of children with cerebral palsy. Conducting a survey assessment of pwMS and their families to identify unmet needs that could be addressed through social prescribing, followed by a trial evaluating its impact on disease outcomes, is a productive next step toward mitigating the influence of SDH and social isolation on therapeutic interventions like aerobic exercise.

## Future Directions and Research Needs

Although social prescribing has existed for decades and is now utilized across several countries, its potential in neurologic diseases remains largely underexplored. Substantial evidence supports the benefits of aerobic exercise for pwMS, yet research on strategies to increase physical activity levels in this population to match those of the general population is lacking. This review highlights the need to move beyond documenting underutilization of exercise in MS care and instead design rigorous studies that test interventions aimed at addressing the psychosocial, environmental, and structural barriers that limit adherence to exercise recommendations from neurologists and PCPs.

This review identifies key evidence gaps in exercise implementation for MS, particularly among those with advanced disability, where guidelines remain incomplete. No studies have evaluated social prescribing in this population. Future research should include large, diverse, longitudinal trials across all disability levels, with priority on advanced MS. Developing and testing MS-specific social prescribing protocols is also essential, with attention to feasibility, cost-effectiveness, and equitable access for underserved groups. To ensure relevance, such interventions should be co-designed with pwMS and their caregivers.

## Conclusion

In summary, structured and safe aerobic exercise programs offer clear benefits for pwMS, alleviating some of the most common and burdensome symptoms, such as fatigue, cognitive challenges, impaired mobility, and mood disturbances, thereby supporting overall quality of life. Despite guideline recommendations from the National Multiple Sclerosis Society, real-world participation remains low, reflecting the unique barriers faced by individuals with a chronic demyelinating disease. Addressing these obstacles requires patient-centered strategies that consider social determinants of health, with social prescribing serving as one possible approach to mitigate these barriers. Future research should focus on scalable interventions to enhance exercise adherence, ensuring that the proven benefits of physical activity are accessible to all people living with MS.

## Key Points

- Multiple sclerosis is the most common cause of nontraumatic disability in young adults with high degree of heterogeneity in clinical symptoms.
- Aerobic exercise is the most effective and versatile intervention to improve quality of life. However, there are significant barriers to exercise, and their interplay with social determinants of health.
- There could be several barriers to exercise, including symptoms of multiple sclerosis, psycho-social factors, socio-economic status leading to limited resources and current healthcare structure, and others.
- Social prescribing can provide both logistical and emotional support to allow the person to engage in the physical activity recommended by the primary care physician or the neurologist.
- It is critical to develop personalized exercise plan tailored to physical and clinical conditions with emphasis on the safety during exercise and prevention of overexertion with the progression of disability.
- Targeted psychological interventions and the integration of cognitive-behavioral strategies into exercise programs would enhance the adherence among depressed patients with multiple sclerosis.
- Further investigations are warranted with carefully designed longitudinal clinical studies in large and diverse population across all disability levels, with priority on advanced multiple sclerosis with attention to feasibility, cost-effectiveness, and equitable access for underserved groups.
